# Endoscopic ultrasound-assisted rendezvous in an intradiverticular papilla: a step-by-step salvage approach

**DOI:** 10.1055/a-2587-9407

**Published:** 2025-05-06

**Authors:** Petr Vanek, Guru Trikudanathan

**Affiliations:** 1 5635 Division of Gastroenterology, Hepatology and Nutrition, University of Minnesota Twin Cities, Minneapolis, United States; 2 48207 Faculty of Medicine and Dentistry, Palacky University Olomouc, Olomouc, Czech Republic; 3 48275 Department of Gastroenterology and Digestive Endoscopy, Masaryk Memorial Cancer Institute, Brno, Czech Republic

An intradiverticular papilla is a significant challenge in pancreatobiliary endoscopy, frequently causing failed conventional ERCP 1 . The prevalence of periampullary diverticula increases with age, reaching up to 65% in the elderly 2 . We present an 81-year-old woman with recurrent abdominal pain and inconclusive imaging suggestive of choledocholithiasis. Two prior ERCP attempts failed due to the papilla’s intradiverticular location ( Fig. 1 ). Endoscopic ultrasound (EUS)-assisted biliary rendezvous (EUS-RV) was pursued as a salvage strategy, providing both conclusive imaging and ductal access in a single-session ( Video 1 ).

**Fig. 1:**
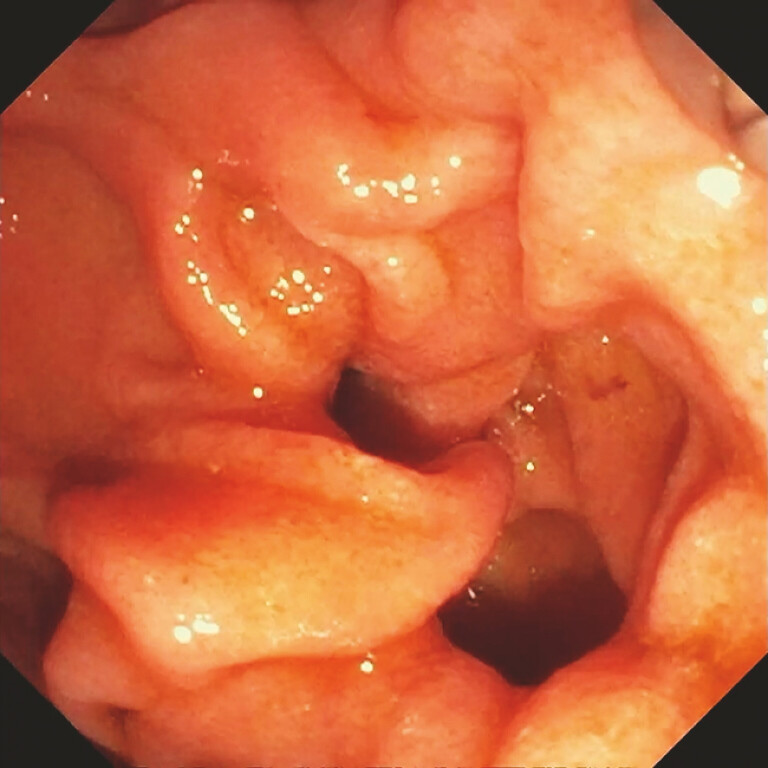
Intradiverticular major papilla visualized endoscopically.

Step-by-step endoscopic ultrasound-assisted rendezvous for choledocholithiasis in the setting of an intradiverticular papilla.Video 1

First, EUS revealed a 9-mm stone in the common hepatic duct with diffuse intra- and extrahepatic dilatation ( Fig. 2 ). After briefly confirming that attempts to locate or cannulate the papilla had failed, a 19-gauge needle was advanced transduodenally into the extrahepatic bile duct under EUS guidance, with proper positioning confirmed via cholangiogram ( Fig. 3 ). A 0.025-inch × 450-cm straight-tip guidewire (VisiGlide, Olympus Corp.) was then navigated antegrade through the ampulla into the duodenum ( Fig. 4 ). The echoendoscope was exchanged for a duodenoscope, preserving wire access. Although initial “along-the-wire” cannulation failed, backloading the wire into the duodenoscope enabled the “over-the-wire” approach, resulting in successful biliary cannulation ( Fig. 5 ). Therapeutic interventions, including stone extraction, were then completed without complications.

**Fig. 2:**
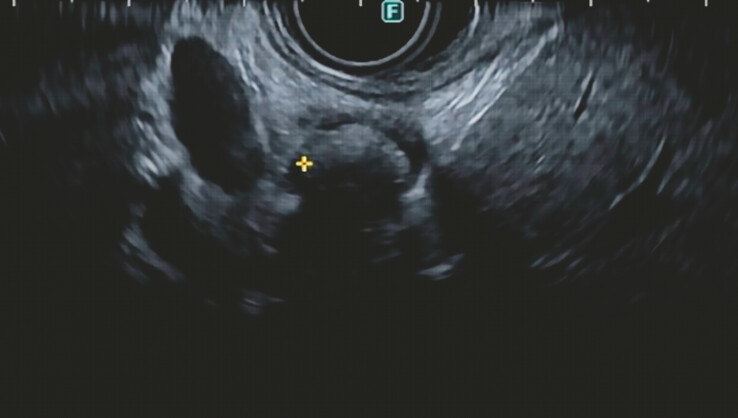
EUS image showing a 9-mm stone lodged in the common hepatic duct near the cystic duct take-off. Abbreviation: EUS, endoscopic ultrasound.

**Fig. 3:**
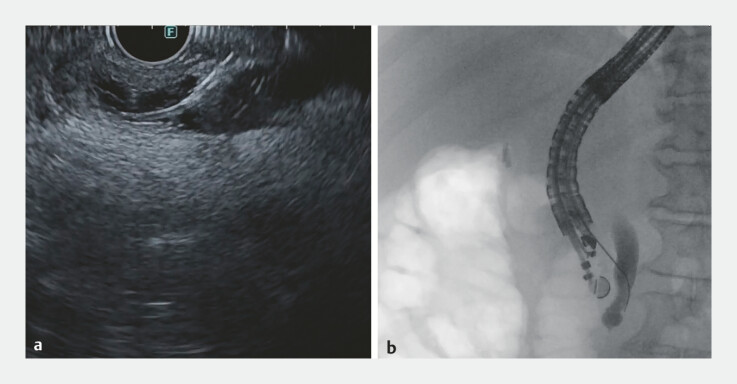
EUS-guided puncture of the extrahepatic bile duct (left); cholangiography confirming correct position of the 19-gauge needle (right). Abbreviation: EUS, endoscopic ultrasound.

**Fig. 4:**
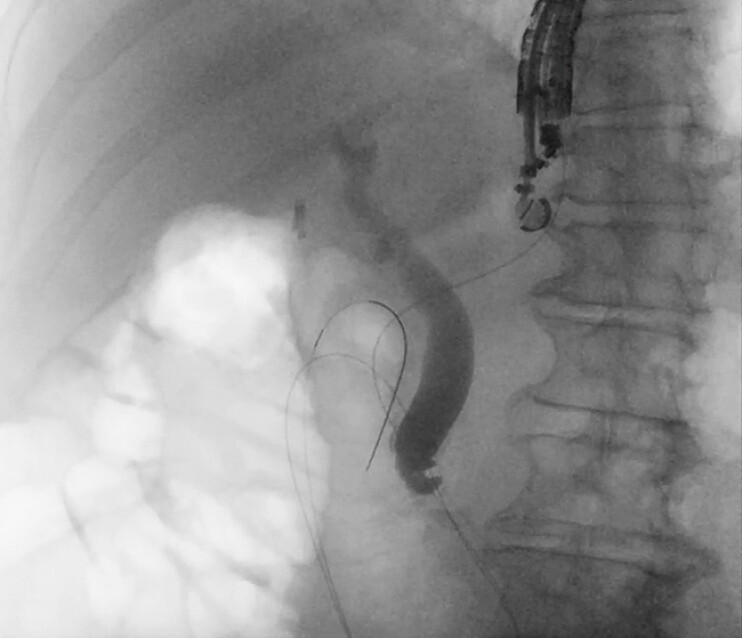
Fluoroscopic view demonstrating the guidewire advanced in an antegrade fashion through the ampulla into the duodenum.

**Fig. 5:**
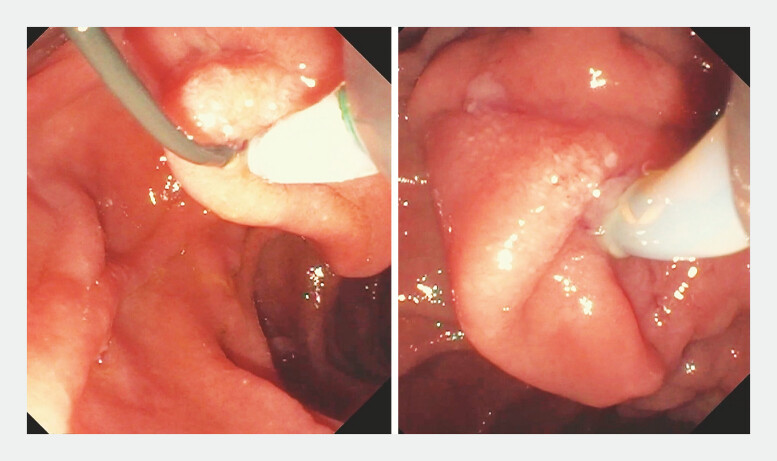
Cannulation attempt using the “along-the-wire” technique (left); successful biliary cannulation using the “over-the-wire” approach after backloading the guidewire into the duodenoscope (right).

Given the lack of comparative data on biliary cannulation methods for intradiverticular papilla, the European Society of Gastrointestinal Endoscopy does not provide a definitive algorithm 1 3 . When ERCP fails, various EUS-guided biliary drainage options may be considered, depending on underlying pathology (benign/malignant) and obstruction level (distal/hilar) 3 . The case highlights a complex intervention where EUS-RV offered both diagnostic confirmation and an alternative route for therapy. Although an established technique, it remains underutilized at most centers 4 . While EUS-RV may seem straightforward, it can be challenging even for advanced endoscopists. This video case provides a procedural blueprint and practical tips for endoscopists with limited exposure to the technique.

Endoscopy_UCTN_Code_TTT_1AR_2AB

## Notes

Endoscopy E-Videos https://eref.thieme.de/e-videos
*E-Videos* is an open access online section of the journal *Endoscopy* , reporting on interesting cases and new techniques in gastroenterological endoscopy. All papers include a high-quality video and are published with a Creative Commons CC-BY license. Endoscopy E-Videos qualify for HINARI discounts and waivers and eligibility is automatically checked during the submission process. We grant 100% waivers to articles whose corresponding authors are based in Group A countries and 50% waivers to those who are based in Group B countries as classified by Research4Life (see: https://www.research4life.org/access/eligibility/ ). This section has its own submission website at https://mc.manuscriptcentral.com/e-videos .
